# OSWRKY114 Negatively Regulates Drought Tolerance by Restricting Stomatal Closure in Rice

**DOI:** 10.3390/plants11151938

**Published:** 2022-07-26

**Authors:** Giha Song, Seungmin Son, Kyong Sil Lee, Yeo Jin Park, Eun Jung Suh, Soo In Lee, Sang Ryeol Park

**Affiliations:** National Institute of Agricultural Sciences, Rural Development Administration, Jeonju 54874, Korea; geometry@korea.kr (G.S.); linewind@korea.kr (S.S.); golderic@naver.com (K.S.L.); jin7188@hanmail.net (Y.J.P.); seji00@korea.kr (E.J.S.); silee@korea.kr (S.I.L.)

**Keywords:** drought, *OsWRKY114*, *PYR/PYL/RCAR*, rice, stomatal closure

## Abstract

The WRKY family of transcription factors plays a pivotal role in plant responses to biotic and abiotic stress. The WRKY Group III transcription factor OsWRKY114 is a positive regulator of innate immunity against *Xanthomonas oryzae* pv. *oryzae*; however, its role in abiotic stress responses is largely unknown. In this study, we showed that the abundant *OsWRKY114* transcripts present in transgenic rice plants are reduced under drought conditions. The overexpression of *OsWRKY114* significantly increased drought sensitivity in rice, which resulted in a lower survival rate after drought stress. Moreover, we showed that stomatal closure, which is a strategy to save water under drought, is restricted in *OsWRKY114*-overexpressing plants compared with wild-type plants. The expression levels of *PYR/PYL/RCAR* genes, such as *OsPYL2* and *OsPYL10* that confer drought tolerance through stomatal closure, were also markedly lower in the *OsWRKY114*-overexpressing plants. Taken together, these results suggest that OsWRKY114 negatively regulates plant tolerance of drought stress via inhibition of stomatal closure, which would otherwise prevent water loss in rice.

## 1. Introduction

Drought stress is the most imperative limiting factor for crop yields and can cause huge economic losses. Global warming caused by fossil fuel use is exacerbating drought damage globally. Therefore, the identification of genes involved in drought responses, and their regulatory mechanisms, is necessary to cope with future crises.

Under water-limiting drought conditions, plants first perceive drought stress in their roots. Several molecular signals, including abscisic acid (ABA), are then produced in the roots and move to the shoots [1]. Finally, ABA is synthesized mainly in the vasculature of the leaves and is delivered to the guard cells via ABA importers, including ATP-binding cassette (ABC) transporter G family members (ABCGs) [2]. In guard cells, the pyrabactin-resistance 1/pyrabactin-resistance like/regulatory component of the ABA receptor (PYR/PYL/RCAR) recognizes ABA and inhibits type 2C protein phosphatases (PP2Cs), which are negative regulators of sucrose non-fermenting 1 (SNF1)-related protein kinases 2 (SnRK2s), thereby activating ABA signal transduction [3]. As a result, the osmotic pressure and volume of guard cells are decreased by the release of anions and K^+^, which stimulates stomatal closure to reduce transpirational water loss [4,5].

The WRKY transcription factor (TF) superfamily, comprising many members, is involved in various plant processes including responses to biotic and abiotic stress [6]. These TFs are classified by their possession of the WRKY domain (WD) consistent with the highly conserved heptad WRKYGQK, known as the WRKY motif, in the N-terminus and a zinc finger domain in the C-terminus [7]. The WRKY TFs are subdivided into three groups: Group I TFs contain two WDs; Group II TFs contain one WD with C_2_H_2_ zinc finger; and Group III TFs contain one WD with C_2_HC zinc finger [8]. Among them, the WRKY Group III TFs are considered the most evolutionarily advanced and adaptable WRKY TFs [9]. Group III TFs participate mainly in innate immunity against various pathogens and function as essential components of not only basal defense but also of systemic acquired resistance [10,11,12]. However, few WRKY Group III TFs involved in abiotic stress are known.

Rice (*Oryza sativa* L.), which is one of the most important monocot crops, has 103 WRKY TFs, including 28 Group III members [13,14]. Among rice WRKY TFs, OsWRKY5, OsWRKY45, and OsWRKY55 decrease drought tolerance, while OsWRKY11, OsWRKY30, and OsWRKY47 increase it [15,16,17,18,19,20]. OsWRKY45, OsWRKY47, and OsWRKY55 are WRKY Group III TFs. OsWRKY45 is a particularly important positive regulator of innate immunity against the rice blast and leaf blight pathogens [21,22]. In addition, OsWRKY45 increases drought tolerance in Arabidopsis (*Arabidopsis thaliana*) but decreases it in rice [15,23]. Therefore, WRKY Group III TFs can play various roles as regulators of both abiotic and biotic stress.

Our previous study showed that OsWRKY114 enhances disease resistance to *Xanthomonas oryzae* pv. *oryzae* (*Xoo*) and directly activates the promoters of *pathogenesis-related* (*PR*) genes such as *OsPR1a* and *chitinase* in rice plants [24]. However, the function of OsWRKY114 in response to abiotic stress remains to be elucidated. In this study, we revealed that OsWRKY114 negatively regulates the drought response in rice via the inhibition of stomatal closure.

## 2. Results

### 2.1. Drought Stress Decreases OSWRKY114 Transcript Levels

**Figure 1:**
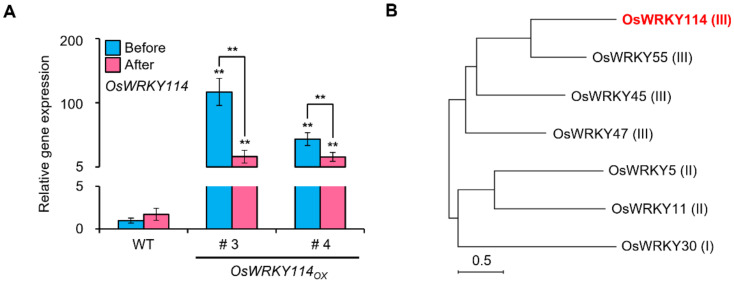
OsWRKY114 expression responds to drought stress. (**A**) Relative transcript levels of *OsWRKY114* in wild-type rice plants (WT) and *OsWRKY114*-overexpressing rice plants (*OsWRKY114_OX_*) before and after drought treatment. Three-week-old rice plants were subjected to drought by withholding water for 10 days. Total RNA was extracted from leaves of rice plants before and after drought treatment. *OsWRKY114* transcripts were analyzed by RT-qPCR. *OsActin* was used as an internal control. Values are expressed as means ± SD. Asterisks indicate values statistically different from the WT before drought treatment and between before and after drought treatment within *OsWRKY114_OX_* plants (** *p* < 0.01). (**B**) Phylogenetic relationship of OsWRKY114 to OsWRKYs regulating drought tolerance in rice. The phylogenic tree was constructed based on the maximum likelihood method using MEGA-X with full-length amino acid sequences. The numerals in parentheses indicate the WRKY group. The bar indicates an evolutionary distance of 0.5 substitutions per nucleotide. All experiments were repeated at least three times with similar results.

To examine whether OsWRKY114 is involved in not only biotic stress but also abiotic stress, we performed RT-qPCR and monitored the transcription level of *OsWRKY114* after drought stress treatment. The transcription level of *OsWRKY114* was similar before and after drought treatment in the wild-type plants (Figure 1A). However, interestingly, the *OsWRKY114* transcript levels in the *OsWRKY114_OX_* plants were significantly lower after drought than before drought (Figure 1A). 

Six OsWRKYs (OsWRKY5, OsWRKY11, OsWRKY30, OsWRKY45, OsWRKY47, and OsWRKY55) are known regulators of drought tolerance in rice [15,16,17,18,19,20]. To examine the relationships among these TFs, we constructed a phylogenetic tree. OsWRKY114 was grouped with WRKY Group III OsWRKY55 that reduces drought tolerance (Figure 1B). Moreover, a previous study showed that OsWRKY55 is one of the closest OsWRKY TFs to OsWRKY114 in amino acid sequences [24]. These results suggest that OsWRKY114 is involved in plant responses to drought stress as a negative regulator.

### 2.2. OSWRKY114 Negatively Regulates Drought Tolerance

**Figure 2:**
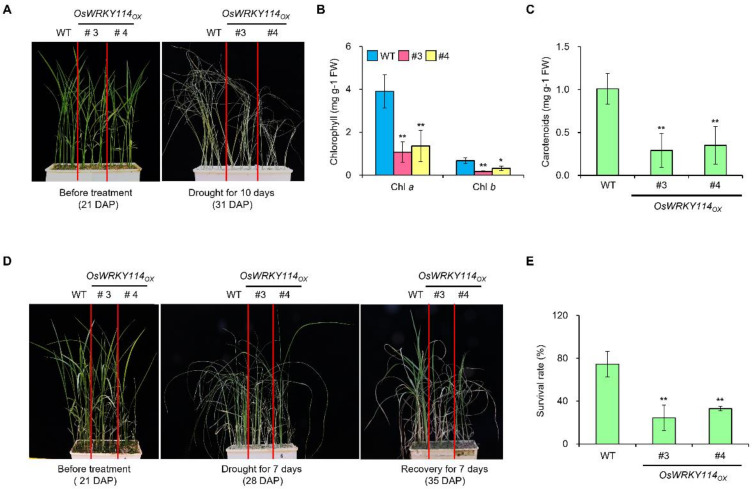
*OsWRKY114* overexpression reduces drought tolerance. (**A**) Drought phenotypes of *OsWRKY114*-overexpressing (*OsWRKY114_OX_*) and wild-type rice plants (WT). Three-week-old rice plants were subjected to drought by withholding water for 10 days. Images were acquired before and after drought treatment (i.e., at 21 and 31 days after planting (DAP), respectively). (**B**,**C**) Plant pigment contents after drought stress. Three-week-old rice plants were treated with drought stress for 10 days and their contents of chlorophyll (**B**) and carotenoid (**C**) were calculated. Values are expressed as means ± SD. Asterisks indicate values statistically different from those of the WT (* *p* < 0.05 and ** *p* < 0.01). (**D**,**E**) Survival rates of drought-treated *OsWRKY114_OX_* and WT plants after re-watering. Three-week-old rice plants were subjected to drought by withholding water. Seven days after drought treatment, they were rewatered and allowed to recover for 7 days. Images were acquired on indicated days (**D**) and the survival rates were measured 7 days after water recovery (**E**). Values are expressed as means ± SD. Asterisks indicate values statistically different from those of the WT (** *p* < 0.01). All experiments were repeated at least three times with similar results (12 replicates for each genotype).

To determine whether OsWRKY114 affects drought tolerance, we subjected *OsWRKY114_OX_* and wild-type plants to drought stress. After drought treatment, almost all the wild-type plants had wilted and the *OsWRKY114_OX_* lines exhibited even more wilting than the wild-type plants (Figure 2A). Since drought stress decreases the contents of plant pigments such as chlorophylls and carotenoids in rice [25], we measured chlorophyll and carotenoid contents to confirm the drought-sensitive phenotype of the *OsWRKY114_OX_* lines. The chlorophyll a, chlorophyll b, and carotenoid contents were significantly lower in the *OsWRKY114_OX_* plants than in the wild-type plants (Figure 2B,C).

To test whether OsWRKY114 also affects the recovery of rice after drought stress, we rewatered *OsWRKY114_OX_* and the wild-type plants after the drought treatment (Figure 2D). After rewatering, the survival rate of the *OsWRKY114_OX_* lines was less than 33%, while that of the wild-type plants was approximately 75% (Figure 2E). Taken together, these results suggest that OsWRKY114 increases drought sensitivity in rice.

### 2.3. Stomatal Closure and Pyl Gene Expression Were Reduced in OSWRKY114-Overexpressing Plants

**Figure 3:**
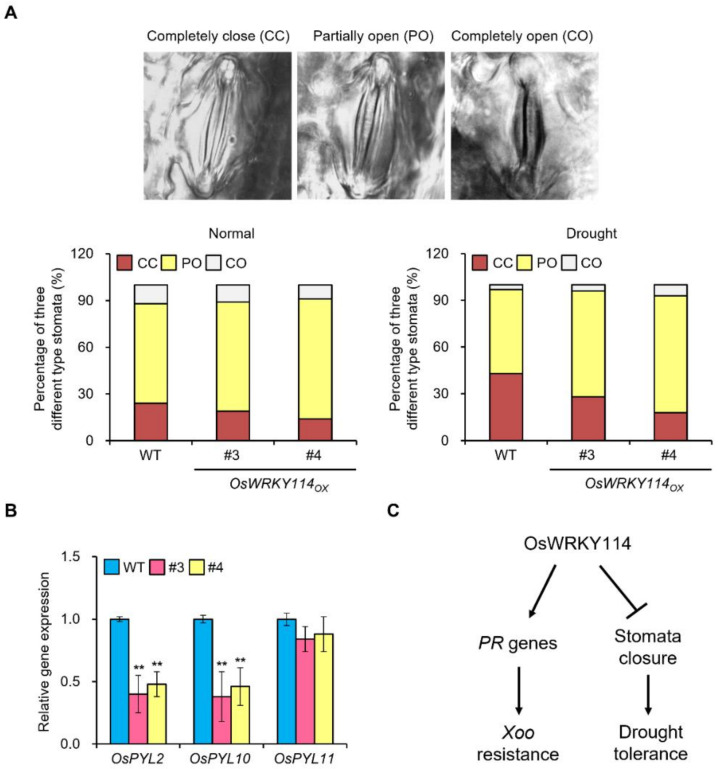
*OsWRKY114* overexpression affects stomatal closure and expression levels of *OsPYL* genes. (**A**) The percentages of three different stoma types in *OsWRKY114*-overexpressing rice lines (*OsWRKY114_OX_*) and wild-type rice plants (WT). Stomatal apertures were observed in the first fully expanded leaves of 3-week-old *OsWRKY114_OX_* and WT plants under normal and drought conditions. (**B**) Relative gene expression levels of *OsPYL* genes. Total RNA was extracted from leaves of 3-week-old *OsWRKY114_OX_* and WT plants. The transcript levels of *OsPYL* genes were analyzed by RT-qPCR. *OsActin* was used as an internal control. Values are expressed as means ± SD. Asterisks indicate values statistically different from those of the WT (** *p* < 0.01). (**C**) A working model of OsWRKY114 involvement in both abiotic and biotic stress responses. All experiments were repeated at least three times and generated similar results.

Stomata play major roles in drought stress through regulating water loss. Thus, we investigated whether OsWRKY114 is associated with stomatal closure. We observed the leaf stomatal apertures of *OsWRKY114_OX_* and wild-type plants under normal and drought conditions. The percentage of completely closed stomata was higher in *OsWRKY114_OX_* and the wild-type plants under the drought condition than under the normal condition (Figure 3A). However, under the drought condition, the percentage of completely closed stomata was lower in the *OsWRKY114_OX_* plants than in the wild-type plants (Figure 3A). Thus, OsWRKY114 inhibits stomatal closure, especially after drought treatment.

To explore the molecular mechanism underlying the negative effect of OsWRKY114 in drought tolerance, we examined the expression levels of *OsPYL* genes conferring drought tolerance via stomatal closure. As a result, we revealed that *OsPYL2* and *OsPYL10* had significantly lower transcript levels in the *OsWRKY114_OX_* lines than in the wild-type plants (Figure 3B). The tissue expression profiles using rice expression databases showed that *OsWRKY114*, *OsPYL2*, and *OsPYL10* are expressed in various tissues abundantly (Supplementary Materials Figure S1). Taken together, these results suggest that OsWRKY114 reduces drought tolerance by the down-regulation of *OsPYL2* and *OsPYL10* transcripts.

## 3. Discussion

Drought is a major environmental stress threatening crop production globally. The severity of drought stress is expected to increase due to climate change. To mitigate the effect of drought on crop production, drought-resistant crops can now be bred through the use of plant breeding technologies based on site-specific nucleases [26]. Therefore, the identification of TFs coordinating the expression of genes involved in drought responses is important for crop improvement.

Previously, we identified WRKY Group III OsWRKY114 as a positive regulator of rice resistance against *Xoo* [24]. To determine whether OsWRKY114 is also involved in abiotic stress, we treated drought stress to *OsWRKY114_OX_*. The transcript level of *OsWRKY114* was significantly reduced in the *OsWRKY114_OX_* lines after drought stress (Figure 1A). Overexpression of *OsWRKY114* indeed decreased rice drought tolerance, while also reducing the plants’ recovery rate after rewatering (Figure 2D,E). Moreover, the percentage of completely closed stomata after drought treatment was lower in *OsWRKY114_OX_* plants than in wild-type plants (Figure 3A).

Plants close stomata to conserve water under drought. Since stomatal closure begins with the recognition of ABA by PYL/RCARs in guard cells, PYL/RCARs play a critical role in the drought response. The rice genome contains 13 *OsPYL* genes [27], and previous studies revealed that the overexpression of *OsPYL2/RCAR9* (Os06g36670), *OsPYL10/RCAR3* (Os02g15640), and *OsPYL11/RCAR5* (Os05g12260) confers drought tolerance in rice. We therefore analyzed the expression levels of *OsPYL* genes in the *OsWRKY114_OX_* lines. Interestingly, the expression levels of *OsPYL2* and *OsPYL10* were lower in the *OsWRKY114_OX_* plants than in the wild-type plants, while expression levels of *OsPYL11* were similar (Figure 3B). Taken together, our results suggest that OsWRKY114 negatively regulates drought tolerance through the inhibition of stomatal closure. Previously, we revealed that OsWRKY114 is a transcriptional activator and directly increases the gene expression of *OsPR1a* and *chitinase* [24]. However, since OsWRKY114 reduced the transcription levels of *OsPYL2* and *OsPYL10*, OsWRKY114 may regulate the *OsPYLs* expression indirectly. The detailed mechanism of how OsWRKY114 inhibits the gene expression should be elucidated in future studies.

In conclusion, our study suggests that OsWRKY114 regulates not only biotic stress but also abiotic stress (Figure 3C). This implies that OsWRKY114 may serve as a component of crosstalk signaling pathways involved in biotic and abiotic responses. This study provides important information for further investigations of OsWRKY114 and plant breeding conferring drought tolerance. 

## 4. Materials and Methods

### 4.1. Plant Material and Growth Conditions

Rice kernels (*Oryza sativa* L. cv. Ilim) were used as the wild type in this study. *OsWRKY114*-overexpressing rice plants (*OsWRKY114_OX_*) were previously generated [24]. Before sowing, all kernels were sterilized in 2% sodium hypochlorite solution for 30 min and rinsed 5 times with sterilized distilled water. The kernels were then incubated in a growth chamber in the dark at 28 °C. The rice plants were grown in square pots (16 × 8 × 7cm) filled with nursery soil consisting of 60% zeolite, 20% diatomite, 19.67% vermiculite, 0.3% fertilizer, and 0.03% pH stabilizer (pH 5.5) in a greenhouse with a 16 h light/8 h dark cycle at 28 °C and 35–40% humidity.

### 4.2. Gene Expression Analysis

The leaves of the plants were frozen and ground to powder under liquid nitrogen in the middle of the photoperiod (12:00). Total RNA was extracted from the leaves using a RNeasy Plant Mini Kit (QIAGEN, Germantown, MD, USA), and then cDNA was synthesized from 1 µg of total RNA using SuperScript III reverse transcriptase (Invitrogen, Waltham, MA, USA) according to the manufacturer’s instructions. Quantitative reverse transcription polymerase chain reaction (RT-qPCR) was performed using specific primers (Table S1). RT-qPCR was conducted on a QuantaStudio 3 PCR System (Thermo Fisher Scientific, Waltham, MA, USA) using SYBR Green Master Mix (Enzynomics, Daejeon, Korea). Gene expression was quantified using the comparative Ct method. *OsActin* was used as an internal control to determine the expression of genes.

### 4.3. Drought Stress Treatment

Drought phenotype analysis was performed as previously described [28] with slight modifications. Briefly, the wild-type and the *OsWRKY114_OX_* plants were grown in soil with 80.2 ± 0.5% water content for 3 weeks. To test the effects of drought, 3-week-old plants were subjected to drought for 10 days by withholding water content to 4.6 ± 0.8%. To measure the plant recovery from drought, 3-week-old plants were subjected to drought for 7 days, and then they were rewatered to 78.8 ± 0.4% water content. The survival rates were recorded after 7 days of rewatering. Images were acquired on indicated days, and representative data are presented.

### 4.4. Chlorophyll and Carotenoid Content Measurement

To measure the total chlorophyll and carotenoid contents, pigments were extracted from equal fresh weights of leaves with 80% acetone solution. The concentration of total chlorophyll was determined using a UV/VIS spectrophotometer (Thermo Fisher Scientific, Waltham, MA, USA) and calculated as previously described [29].

### 4.5. Physiological Measurement

Different types of stomata were analyzed as previously described [28]. The first fully expanded leaves of 3-week-old wild-type and *OsWRKY114_OX_* plants were used. For drought treatment, the 3-week-old plants were subjected to drought for 10 days by withholding water, and their first fully expanded leaves were used. The stomata were observed using a TCS SP8 confocal microscope (Leica, Wetzlar, Germany).

### 4.6. Statistical Analysis

All experiments were repeated at least three times and the data were analyzed using *t*-tests with GraphPad Prism 8.0.2 software.

## Data Availability

The data presented in this study are available in the article or the Supplementary Materials.

## Supplementary Materials

The following supporting information can be downloaded at: https://www.mdpi.com/article/10.3390/plants11151938/s1, Figure S1: Expression profiles of OsWRKY114, OsPYL2, and OsPYL10 in various tissues. Expression profiling analysis was performed using online rice expression databases of Genevestigator (https://genevestigator.com/ accessed on 26 May 2022); Table S1: Sequence of primers used in this study.
