# Supplementary material for: OSWRKY114 Negatively Regulates Drought Tolerance by Restricting Stomatal Closure in Rice
Source: Plants (Basel). 2022 Jul 26;11(15):1938. doi: 10.3390/plants11151938 (PMC9331222; doi:10.3390/plants11151938)
Supplement: Supplementary file 1 [file plants-11-01938-s001.zip › plants-1794181-supplementary-update.pdf]

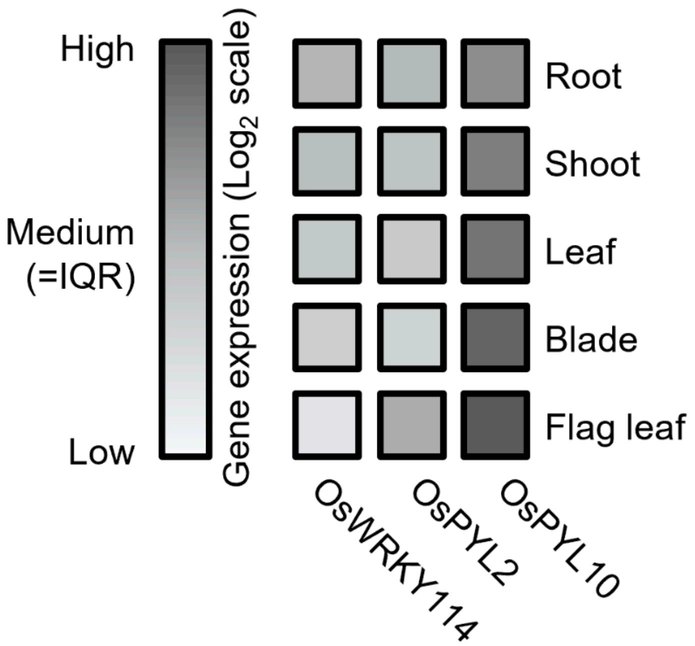

Figure S1. Expression profiles of *OsWRKY114*, *OsPYL2*, and *OsPYL10* in various tissues. Expression profiling analysis was performed using online rice expression databases of Genevestigator (https://genevestigator.com/).

Gene	Primer sequence	Purpose
OsWRKY114	F: CACCCATGTTCCAAGTGACA	RT-qPCR
	R: ATCGTCAGGGTGACCATTTG	RT-qPCR
OsPYL2	F: CACCTCCGTCACCGAGTTCCA	RT-qPCR
	R: ATTGACGAGGCCTCTCCTAGTCG	RT-qPCR
OsPYL10	F: CAAGGATGAGACATGCTACTTCGT	RT-qPCR
	R: GAACCTAAGGGCTCCATTGGAG	RT-qPCR
OsPYL11	F: CTCATCCATCGTAACTGTCCATC	RT-qPCR
	R: GTGATGACCCTACTGTTCAAGTG	RT-qPCR
OsActin	F: CATTGGTGCTGAGCGTTTCC	RT-qPCR
	R: CTCCTTGCTCATCCTGTCAGC	RT-qPCR

Table S1. Sequence of primers used in this study
